# Supplementary material for: Unraveling the Genomic Potential of the Thermophilic Bacterium Anoxybacillus flavithermus from an Antarctic Geothermal Environment
Source: Microorganisms. 2022 Aug 19;10(8):1673. doi: 10.3390/microorganisms10081673 (PMC9413872; doi:10.3390/microorganisms10081673)
Supplement: Supplementary file 1 [file microorganisms-10-01673-s001.zip › microorganisms-1804505-supplementary-proof_JS.pdf]

Supplementary Material

Supplementary Figures

a

BCG	Strains: LAT_11	LAT_26	LAT_27	LAT_31	LAT_33	LAT_35	LAT_38
T3PKS	1	2	1	1	1	1	1
NRPS	0	0	1	0	0	0	0
Betalactone-NRPS	1	1	1	1	1	1	1
Total of regions	2	3	3	2	2	2	2

Number of BCG
0
1
2

b

Prophages	LAT_11	LAT_26	LAT_27	LAT_31	LAT_33	LAT_35	LAT_38
Intact	0	2	0	0	0	0	3
Incomplete	2	3	1	2	2	2	2
Total of regions	2	5	1	2	2	2	5

Figure S1. a) Biosynthetic gene clusters (BGCs) observed in *Anoxybacillus* strains using antiSMASH 5.0. Blank boxes indicate the absence of BGCs, light pink boxes indicate one BGC, and dark pink boxes indicate two related BGCs. b) Prophages identified in the strains using PHASTER. Green boxes indicate intact prophages, and pink boxes indicate questionable prophages.

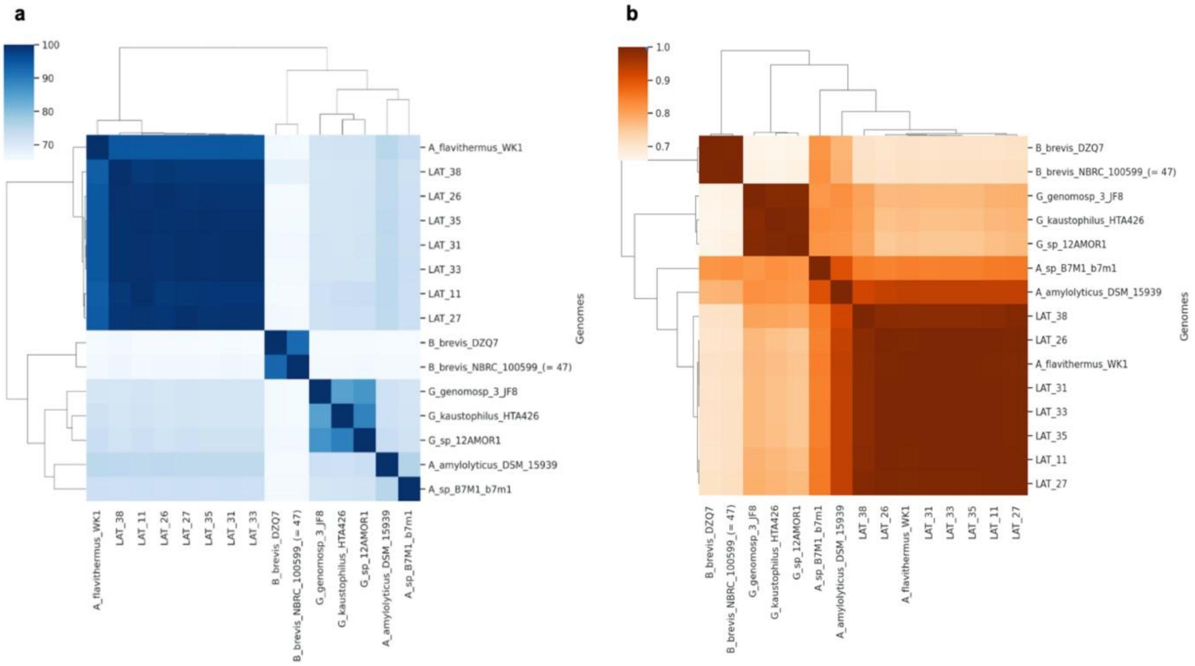

Figure S2. Genome comparison of different *Anoxybacillus* strains and Bacillaceae members based on ANI and TETRA values (%). Based on the ANI values, the strains were grouped into two clusters; the Antarctic strains clustered with *A. flavithermus* WK1 (a). The same pattern was observed using TETRA values (b).

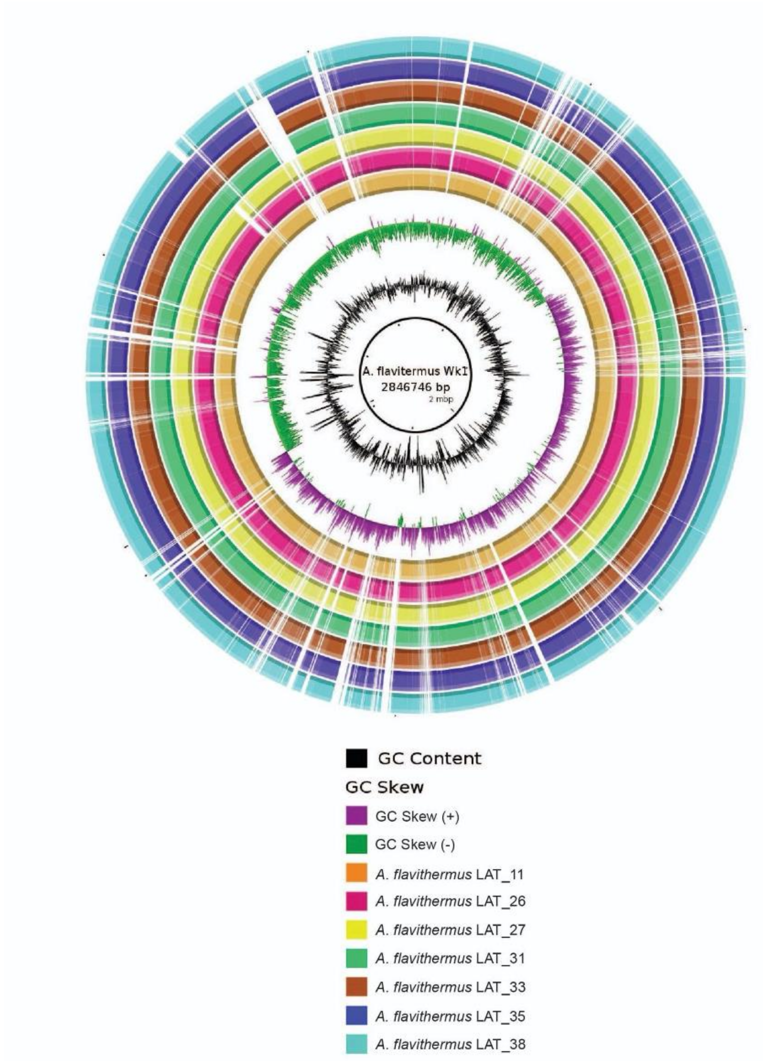

Figure S3. Circular visualization generated by BRIG for comparing the genomes of *Anoxybacillus* strains using *A. flavithermus* WK1 (complete genome) as the reference strain. The inner black circle contains the reference genome, and the intensity of each color indicates the similarity between the strains.

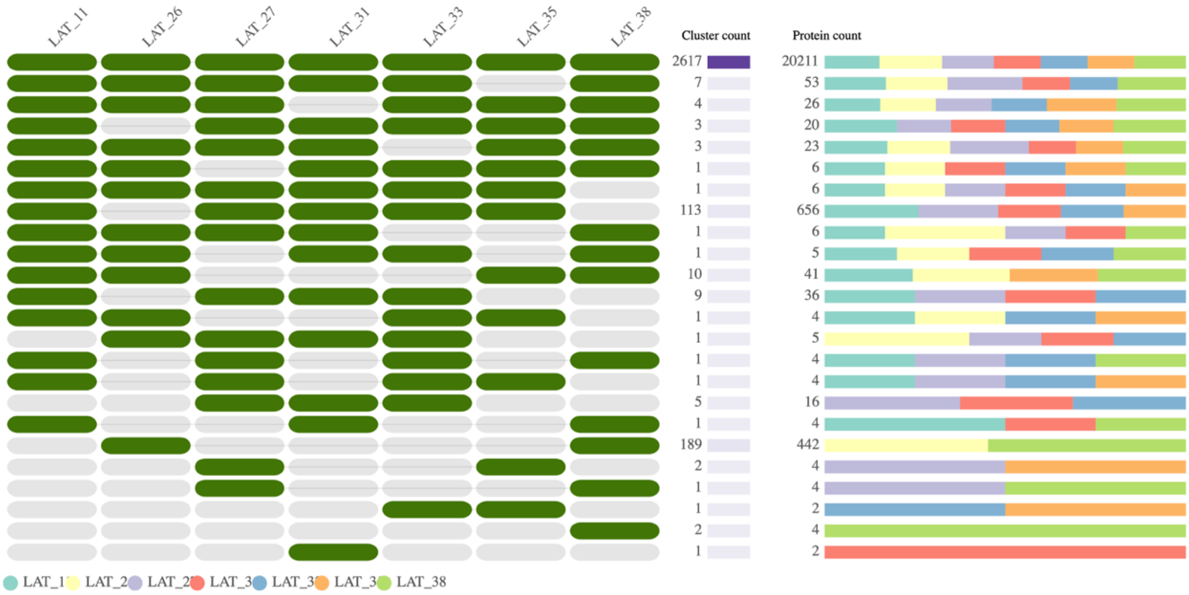

Figure S4. Orthologous groups belonging to the seven *Anoxybacillus* strains analyzed in the present study. From left to right: The orthologous groups present in the genomes; dark green color represents the presence of orthologous groups in each strain, while light gray color represents their absence. Number of protein orthologous groups shared in the genomes, marked with dark lilac in the preceding columns "Cluster count." Total number of proteins (Protein count column) recorded in the shared orthologous groups; the colors represent the proportion of proteins belonging to each strain (light blue = LAT_11; yellow = LAT_26; lilac = LAT_27; red = LAT_31; blue = LAT_33; orange = LAT_35; and green = LAT_38).

2 Supplementary Tables

Table S1. Published data retrieved from databases used in the systematic review of the genus *Anoxybacillus*

Authors	Year	Reference	Link
Acer et al. [82]	2015	Acer Ö, Bekler FM, Pirinççioğlu H, Güven RG, Güven K. Purification and Characterization of Thermostable and Detergent-Stable α-Amylase from Anoxybacillus sp. AH1. Food Technol Biotechnol. **2016,** *54*, 70-77.	https://pubmed.ncbi.nlm.nih.gov/27904395/. accessed on 30 June 2021.
Al-Kahem Al-Balawi Al-balawi et al. [83]	2017	Al-Kahem Al-Balawi TH, Wood AL, Solis A, Cooper T, Barabote RD. Anoxybacillus sp. Strain UARK-01, a New Thermophilic Soil Bacterium with Hyperthermostable Alkaline Laccase Activity. Curr Microbiol. **2017**, *74*, 762-771.	https://pubmed.ncbi.nlm.nih.gov/28389772/. accessed on 30 June 2021.
Atanassova et al. [84]	2008	Atanassova M, Derekova A, Mandeva R, Sjøholm C, Kambourova M. Anoxybacillus bogrovensis sp. nov., a novel thermophilic bacterium isolated from a hot spring in Dolni Bogrov, Bulgaria. Int J Syst Evol Microbiol. **2008**, *58*, 2359-62.	https://pubmed.ncbi.nlm.nih.gov/18842856/. accessed on 30 June 2021.
Belduz et al. [85]	2003	Belduz AO, Dulger S, Demirbag Z. Anoxybacillus gonensis sp. nov., a moderately thermophilic, xylose-utilizing, endospore-forming bacterium. Int J Syst Evol Microbiol. **2003**, *53*, 1315-1320.	https://pubmed.ncbi.nlm.nih.gov/13130012/. accessed on 30 June 2021.
Chai et al. [86]	2001	Chai YY, Kahar UM, Md Salleh M, Md Illias R, Goh KM. Isolation and characterization of pullulan-degrading Anoxybacillus species isolated from Malaysian hot springs. Environ Technol. **2012**, *33*, 1231-8.	https://pubmed.ncbi.nlm.nih.gov/22856294/. accessed on 30 June 2021.
Chan et al. [77]	2016	Chan CS, Sin LL, Chan KG, Shamsir MS, Manan FA, Sani RK, Goh KM. Characterization of a glucose-tolerant β-glucosidase from Anoxybacillus sp. DT3-1. Biotechnol Biofuels. **2016**, *9*, 174.	https://pubmed.ncbi.nlm.nih.gov/27555880/. accessed on 30 June 2021.
Chen et al. [87]	2004	Chen XG, Stabnikova O, Tay JH, Wang JY, Tay ST. Thermoactive extracellular proteases of Geobacillus caldoproteolyticus, sp. nov., from sewage sludge. Extremophiles. **2004**, *8*, 489-98.	https://pubmed.ncbi.nlm.nih.gov/15322950/. accessed on 30 June 2021.
Chen et al. [88]	2015	Chen J, Zheng J, Li Y, Hao HH, Chen JM. Characteristics of a novel thermophilic heterotrophic bacterium, Anoxybacillus contaminans HA, for nitrification-aerobic denitrification. Appl Microbiol Biotechnol. **2015**, *99*, 10695-702.	https://pubmed.ncbi.nlm.nih.gov/26293335/. accessed on 30 June 2021.
Cheng et al. [89]	2021	Cheng JH, Wang Y, Zhang XY, Sun ML, Zhang X, Song XY, Zhang YZ, Zhang Y, Chen XL. Characterization and Diversity Analysis of the Extracellular Proteases of Thermophilic Anoxybacillus	https://pubmed.ncbi.nlm.nih.gov/33796092/. accessed on 30 June 2021.

		caldiproteolyticus 1A02591 From Deep-Sea Hydrothermal Vent Sediment. Front Microbiol. **2021**, *12*, 643508.	
Cihan et al. [90]	2011	Cihan AC, Ozcan B, Cokmus C. Anoxybacillus salavatliensis sp. nov., an α-glucosidase producing, thermophilic bacterium isolated from Salavatli, Turkey. J Basic Microbiol. 2011 Apr;51(2):136-46.	https://pubmed.ncbi.nlm.nih.gov/21077116/. accessed on 30 June 2021.
Cihan et al. [91]	2014	Cihan AC, Cokmus C, Koc M, Ozcan B. Anoxybacillus calidus sp. nov., a thermophilic bacterium isolated from soil near a thermal power plant. Int J Syst Evol Microbiol. 2014 Jan;64(Pt 1):211-219. doi: 10.1099/ijs.0.056549-0. Epub 2013 Sep 19. Erratum in: Int J Syst Evol Microbiol. **2014**, *64*, 2926. Erratum in: Int J Syst Evol Microbiol. 2014 Jul;64(Pt 7):2508.	https://pubmed.ncbi.nlm.nih.gov/24052627/. accessed on 30 June 2021.
Clerck et al. [92]	2004	Clerck E, Rodríguez-Díaz M, Vanhoutte T, Heyrman J, Logan NA, De Vos P. Anoxybacillus contaminans sp. nov. and Bacillus gelatini sp. nov., isolated from contaminated gelatin batches. Int J Syst Evol Microbiol. **2004**, *54*, 941-946.	https://pubmed.ncbi.nlm.nih.gov/15143046/. accessed on 30 June 2021.
Coorevits et al. [93]	2012	Coorevits A, Dinsdale AE, Halket G, Lebbe L, De Vos P, Van Landschoot A, Logan NA. Taxonomic revision of the genus Geobacillus: emendation of Geobacillus, G. stearothermophilus, G. jurassicus, G. toebii, G. thermodenitrificans and G. thermoglucosidans (nom. corrig., formerly 'thermoglucosidasius'); transfer of Bacillus thermantarcticus to the genus as G. thermantarcticus comb. nov.; proposal of Caldibacillus debilis gen. nov., comb. nov.; transfer of G. tepidamans to Anoxybacillus as A. tepidamans comb. nov.; and proposal of Anoxybacillus caldiproteolyticus sp. nov. Int J Syst Evol Microbiol. **2012**, *62*, 1470-1485.	https://pubmed.ncbi.nlm.nih.gov/21856988/. accessed on 30 June 2021.
Deep et al. [94]	2013	Deep K, Poddar A, Das SK. Anoxybacillus suryakundensis sp. nov, a moderately thermophilic, alkalitolerant bacterium isolated from hot spring at Jharkhand, India. PLoS One. **2013**, *8*, e85493. doi: 10.1371/journal.pone.0085493. PMID: 24376881; PMCID: PMC3869905.	https://pubmed.ncbi.nlm.nih.gov/24376881/. accessed on 30 June 2021.
Derekova et al. [95]	2007	Derekova A, Sjøholm C, Mandeva R, Kambourova M. Anoxybacillus rupiensis sp. Nov., a novel thermophilic bacterium isolated from Rupi basin (Bulgaria). Extremophiles. **2007**, *11*, 577-83. doi: 10.1007/s00792-007-0071-4. Epub 2007 May 16. PMID: 17505776.	https://pubmed.ncbi.nlm.nih.gov/17505776/. accessed on 30 June 2021.
Dulger et al. [96]	2004	Dulger S, Demirbag Z, Belduz AO. Anoxybacillus ayderensis sp. nov. and Anoxybacillus kestanbolensis sp. nov. Int J Syst Evol Microbiol. **2004**, *54*, 1499-1503. doi: 10.1099/ijs.0.02863-0. PMID: 15388701.	https://pubmed.ncbi.nlm.nih.gov/15388701/. accessed on 30 June 2021.

Filippidou et al. [97]	2016	Filippidou S, Jaussi M, Junier T, Wunderlin T, Jeanneret N, Palmieri F, Palmieri I, Roussel-Delif L, Vieth-Hillebrand A, Vetter A, Chain PS, Regenspurg S, Junier P. Anoxybacillusgeothermalis sp. nov., a facultatively anaerobic, endospore-forming bacterium isolated from mineral deposits in a geothermal station. Int J Syst Evol Microbiol. **2016**, *66*, 2944-2951. doi: 10.1099/ijsem.0.001125. Epub 2016 Apr 28. PMID: 27126386.	https://pubmed.ncbi.nlm.nih.gov/27126386/. accessed on 30 June 2021.
Gao et al. [98]	2010	Gao Y, Dai J, Peng H, Liu Y, Xu T. Isolation and characterization of a novel organic solvent-tolerant Anoxybacillus sp. PGDY12, a thermophilic Gram-positive bacterium. J Appl Microbiol. **2011**, *110*, 472-8. doi: 10.1111/j.1365-2672.2010.04903.x. Epub 2010 Dec 13. PMID: 21155953.	https://pubmed.ncbi.nlm.nih.gov/21155953/. accessed on 30 June 2021.
Gul-Guven et al. [99]	2008	Gul-Guven R, Guven K, Poli A, Nicolaus B. Anoxybacillus kamchatkensis subsp. asaccharedens subsp. nov., a thermophilic bacterium isolated from a hot spring in Batman. J Gen Appl Microbiol. **2008**, *54*, 327-34. doi: 10.2323/jgam.54.327. PMID: 19164875.	https://pubmed.ncbi.nlm.nih.gov/19164875/. accessed on 30 June 2021.
Heinen at al. [2]	1982	Heinen W, Lauwers AM, Mulders JW. Bacillus flavothermus, a newly isolated facultative thermophile. Antonie Van Leeuwenhoek. **1982**, *48*, 265-72. doi: 10.1007/BF00400386. PMID: 7125637.	https://pubmed.ncbi.nlm.nih.gov/7125637/. accessed on 30 June 2021.
Inan et al. [100]	2012	Inan K, Belduz AO, Canakci S. Anoxybacillus kaynarcensis sp. nov., a moderately thermophilic, xylanase producing bacterium. J Basic Microbiol. **2013**, *53*, 410-9. doi: 10.1002/jobm.201100638. Epub 2012 Jun 26. PMID: 22736500.	https://pubmed.ncbi.nlm.nih.gov/22736500/. accessed on 30 June 2021.
Kacagan et al. [101]	2008	Kacagan, Murat & Canakci, Sabriye & Sandalli, Cemal & Inan Bektas, Kadriye & Colak, Dilsat & Belduz, Ali. Characterization of a xylanase from a thermophilic strain of Anoxybacillus pushchinoensis A8. Biologia. **2008**, *63*, doi: 599-606. 10.2478/s11756-008-0134-8.	https://www.researchgate.net/publication/225168883_Characterization_of_a_xylanase_from_a_thermophilic_strain_of_Anoxybacillus_pushchinoensis_A8. accessed on 30 June 2021.
Kevbrin et al. [102]	2005	Kevbrin VV, Zengler K, Lysenko AM, Wiegel J. Anoxybacillus kamchatkensis sp. nov., a novel thermophilic facultative aerobic bacterium with a broad pH optimum from the Geyser valley, Kamchatka. Extremophiles. **2005**, *9*, 391-8. doi: 10.1007/s00792-005-0479-7. Epub 2005 Sep 3. PMID: 16142505.	https://pubmed.ncbi.nlm.nih.gov/16142505/. accessed on 30 June 2021.
Khan et al. [103]	2018	Khan IU, Habib N, Xiao M, Devi AM, Habib M, Hejazi MS, Salam N, Zhi XY, Li WJ. Anoxybacillus sediminis sp. nov., a novel moderately thermophilic bacterium isolated from a hot spring. Antonie Van Leeuwenhoek. **2018**, *111*, 2275-2282. doi: 10.1007/s10482-018-1118-5. Epub 2018 Jun 21. PMID: 29931494.	https://pubmed.ncbi.nlm.nih.gov/29931494/. accessed on 30 June 2021.

Khanna et al. [104]	2021	Khanna K, Mishra KP, Chanda S, Ganju L, Singh SB, Kumar B. Effect of Synbiotics on Amelioration of Intestinal Inflammation Under Hypobaric Hypoxia. High Alt Med Biol. **2021**, *22*, 32-44.	https://www.liebertpub.com/doi/10.1089/ham.2020.0062?url_ver=Z39.88-2003&rfr_id=ori:rid:crossref.org&rfr_dat=cr_pub%20%200pubmed. accessed on 30 June 2021.
Lee et al. [105]	2012	Lee, Sang-Jae & Lee, Yong-Jik & Ryu, Naeun & Park, Seulki & Jeong, Haeyoung & Lee, Sang Jun & Kim, Byoung-Chan & Lee, Dong-Woo & Lee, Han-Seung. Draft Genome Sequence of the Thermophilic Bacterium Anoxybacillus kamchatkensis G10. Journal of bacteriology. **2012**, *194*, 6684-5. 10.1128/JB.01877-12.	https://www.researchgate.net/publication/233396557_Draft_Genome_Sequence_of_the_Thermophilic_Bacterium_Anoxybacillus_kamchatkensis_G10. accessed on 30 June 2021.
Matpan-Bekler e Güven [106]	2014	Matpan-Bekler, Fatma & Guven, Kemal. (2014). Isolation and production of thermostable α-amylase from thermophilic Anoxybacillus sp. KP1 from Diyadin hot spring in Ağri, Turkey. Biologia. **2014**, *69*, 419-427.	https://www.researchgate.net/publication/260450268_Isolation_and_production_of_thermostable_a-amylase_from_thermophilic_Anoxybacillus_sp_KP1_from_Diyadin_hot_spring_in_Agri_Turkey. accessed on 30 June 2021.
Matpan-Bekler et al. [107]	2018	Matpan-Bekler, Fatma & Yalaz, Secil & Guven, Kemal. Molecular characterisation and numerical analysis of novel moderately thermophile Anoxybacillus sp. FMB1. Romanian Biotechnological Letters. **2018**, *23*,13964-13975.	https://www.researchgate.net/publication/326416833_Molecular_characterisation_and_numerical_analysis_of_novel_moderately_thermophile_Anoxybacillus_sp_FMB1. accessed on 30 June 2021.
Mittal at el. [108]	2017	Mittal, Parul & Saxena, Rituja & Sharma, Vineet. Draft Genome Sequence of Anoxybacillus mongoliensis Strain MB4, a Sulfur-Utilizing Aerobic Thermophile Isolated from a Hot Spring in Tattapani, Central India. Genome Announcements. **2017,** *5*, e01709-16. 10.1128/genomeA.01709-16.	https://www.researchgate.net/publication/314190462_Draft_Genome_Sequence_of_Anoxybacillus_mongoliensis_Strain_MB4_a_Sulfur-Utilizing_Aerobic_Thermophile_Isolated_from_a_Hot_Spring_in_Tattapani_Central_India. accessed on 30 June 2021.
Najar et al. [109]	2018	Najar IN, Sherpa MT, Das S, Das S, Thakur N. Microbial ecology of two hot springs of Sikkim: Predominate population and geochemistry. Science of the Total Environment **2018**, *637-638*, 730-745.	https://www.sciencedirect.com/science/article/pii/S0048969718316693. accessed on 30 June 2021.
Namsaraev et al. [110]	2010	Namsaraev ZB, Babasanova OB, Dunaevsky YE, Akimov VN, Barkhutova DD, Gorlenko VM, Namsaraev BB. Anoxybacillus mongoliensis sp. nov., a novel thermophilic proteinase producing bacterium isolated from alkaline hot spring, central Mongolia. Mikrobiologiia. **2010**, *79*, 516-23. PMID: 21058505.	https://pubmed.ncbi.nlm.nih.gov/21058505/. accessed on 30 June 2021.
Ottesen et al. [111]	2016	Ottesen A, Ramachandran P, Reed E, White JR, Hasan N, Subramanian P, Ryan G, Jarvis K, Grim C, Daquiqan N, Hanes D, Allard M, Colwell R, Brown E, Chen Y. Enrichment dynamics of Listeria monocytogenes and the associated microbiome from naturally contaminated ice cream linked to a listeriosis outbreak. BMC Microbiol. **2016**, *16*, 275.	https://bmcmicrobiol.biomedcentral.com/articles/10.1186/s12866-016-0894-1. accessed on 30 June 2021.

Patel [112]	2015	Patel BK. Draft Genome Sequence of Anoxybacillus Strain BCO1, Isolated from a Thermophilic Microbial Mat Colonizing the Outflow of a Bore Well of the Great Artesian Basin of Australia. Genome Announc. **2015**, *3*, e01547-14. doi: 10.1128/genomeA.01547-14.	https://pubmed.ncbi.nlm.nih.gov/25676761/. accessed on 30 June 2021.
Pikuta et al. [1]	2000	Pikuta E, Lysenko A, Chuvilskaya N, Mendrock U, Hippe H, Suzina N, Nikitin D, Osipov G, Laurinavichius K. Anoxybacillus pushchinensis gen. nov., sp. nov., a novel anaerobic, alkaliphilic, moderately thermophilic bacterium from manure, and description of Anoxybacillus flavitherms comb. nov. Int J Syst Evol Microbiol. **2000**, *50*, 2109-2117.	https://pubmed.ncbi.nlm.nih.gov/11155986/. accessed on 30 June 2021.
Poli et al. [10]	2006	Poli A, Esposito E, Lama L, Orlando P, Nicolaus G, de Appolonia F, Gambacorta A, Nicolaus B. Anoxybacillus amylolyticus sp. nov., a thermophilic amylase producing bacterium isolated from Mount Rittmann (Antarctica). Syst Appl Microbiol. **2006**, *29*, 300-7.	https://pubmed.ncbi.nlm.nih.gov/16682297/. accessed on 30 June 2021.
Poli et al. [60]	2009	Poli A, Romano I, Cordella P, Orlando P, Nicolaus B, Ceschi Berrini C. Anoxybacillus thermarum sp. nov., a novel thermophilic bacterium isolated from thermal mud in Euganean hot springs, Abano Terme, Italy. Extremophiles. **2009**, *13*, 867-74.	https://pubmed.ncbi.nlm.nih.gov/19710998/. accessed on 30 June 2021.
Reis et al. [113]	2020	Reis SVD, Beys-da-Silva WO, Tirloni L, Santi L, Seixas A, Termignoni C, Silva MVD, Macedo AJ. The extremophile Anoxybacillus sp. PC2 isolated from Brazilian semiarid region (Caatinga) produces a thermostable keratinase. J Basic Microbiol. **2020**, *60*, 809-815.	https://pubmed.ncbi.nlm.nih.gov/32602226/. accessed on 30 June 2021.
Sahm et al. [114]	2013	Sahm K, John P, Nacke H, Wemheuer B, Grote R, Daniel R, Antranikian G. High abundance of heterotrophic prokaryotes in hydrothermal springs of the Azores as revealed by a network of 16S rRNA gene-based methods. Extremophiles. **2013**, *17*, 649-62.	https://link.springer.com/article/10.1007/s00792-013-0548-2. accessed on 30 June 2021.
Salam and Obayori [115]	2020	Salam LB, Obayori OS. Remarkable shift in structural and functional properties of an animal charcoal-polluted soil accentuated by inorganic nutrient amendment. J Genet Eng Biotechnol. **2020**, *18*, 70.	https://www.ncbi.nlm.nih.gov/pmc/articles/PMC7658278/. accessed on 30 June 2021.
Wu et al. [116]	2012	Wu S, Wang G, Angert ER, Wang W, Li W, Zou H. Composition, diversity, and origin of the bacterial community in grass carp intestine. PLoS One. **2012**, *7*, e30440.	https://journals.plos.org/plosone/article?id=10.1371/journal.pone.0030440. accessed on 30 June 2021.
Xia et al. [117]	2015	Xia, Wenjie and Dong, Hao and Zheng, Chenggang and Cui, Qingfeng and He, Panqing and Tang, Yongchun. Hydrocarbon degradation by a newly isolated thermophilic Anoxybacillus sp. with bioemulsifier production and new alkB genes. RSC Adv. **2015**, *5*, 102367-102377. The Royal Society of Chemistry.	https://pubs.rsc.org/en/content/articlelanding/2015/ra/c5ra17137g#!divAbstract. accessed on 30 June 2021.

Yadav et al. [118]	2018	Yadav P, Maharjan J, Korpole S, Prasad GS, Sahni G, Bhattarai T and Sreerama L. Production, Purification, and Characterization of Thermostable Alkaline Xylanase From Anoxybacillus kamchatkensis NASTPD13. Front. Bioeng. Biotechnol. **2018,** *6*, 65.	https://www.frontiersin.org/articles/10.3389/fbioe.2018.00065/full. accessed on 30 June 2021.
Yumoto et al. [119]	2004	Yumoto I, Hirota K, Kawahara T, Nodasaka Y, Okuyama H, Matsuyama H, Yokota Y, Nakajima K, Hoshino T. Anoxybacillus voinovskiensis sp. nov., a moderately thermophilic bacterium from a hot spring in Kamchatka. Int J Syst Evol Microbiol. **2004**, *54*, 1239-1242.	https://pubmed.ncbi.nlm.nih.gov/15280298/. accessed on 30 June 2021.
Zhang et al. [120]	2011	Zhang CM, Huang XW, Pan WZ, Zhang J, Wei KB, Klenk HP, Tang SK, Li WJ, Zhang KQ. Anoxybacillus tengchongensis sp. nov. and Anoxybacillus eryuanensis sp. nov., facultatively anaerobic, alkalitolerant bacteria from hot springs. Int J Syst Evol Microbiol. **2011,** *61*, 118-122.	https://pubmed.ncbi.nlm.nih.gov/20173008/. accessed on 30 June 2021.
Zhang et al. [121]	2013	Zhang XQ, Zhang ZL, Wu N, Zhu XF, Wu M. Anoxybacillus vitaminiphilus sp. nov., a strictly aerobic and moderately thermophilic bacterium isolated from a hot spring. Int J Syst Evol Microbiol. **2013**, *63*, 4064-4071.	https://pubmed.ncbi.nlm.nih.gov/23728374/. accessed on 30 June 2021.
Zhao et al. [122]	2017	Zhao C, Chu Y, Li Y, Yang C, Chen Y, Wang X, Liu B. High-throughput pyrosequencing used for the discovery of a novel cellulase from a thermophilic cellulose-degrading microbial consortium. Biotechnol Lett. **2017**, *39*, 123-131.	https://link.springer.com/article/10.1007/s10529-016-2224-y. accessed on 30 June 2021.

Table S2. List of described species of the genus *Anoxybacillus* and their characteristics (identification, isolation, and growth)

Reference	Species	Location	Source	Substrate	Culture medium	Growth condition*	Temperature range	Optimal temperature	pH range	Optimal pH	Genome size	%GC	Accession number
Pikuta et al. [1]	*A. pushchinoensis* K1	Moscow, Russia	Fertilizer samples	Manure samples	Anaerobic basal medium with yeast extract and glucose as carbon source, added after sterilization	An/F. a	37–66°C	62°C	8.0–10.5	9.5–9.7	-	42.20%	NR_037100
Heinen et al. [2]	*A. flavithermus*	North Island, New Zealand	Hot spring	Rock surface grit	Bouillon agar with 5% casein	F. a	30–72°C	60–65°C	5.5–9.0	7.0	-	61%	-
Belduz et al. [85]	*A. gonensis* G2	Balikesir, Turkey	Hot spring	Mud and water	Nutrient broth for enrichment	F. an	40–70°C	55–60°C	6.0–10.0	7.5–8.0	-	57%	NR_025667
Dulger et al. [96]	*A. ayderensis* AB4	Rize, Turkey	Hot spring	Water	Nutrient broth for enrichment	F. an	40–70°C	50°C	6.0–11.0	7.5–8.5	-	54%	AF001963
Chen et al. [88]	*A. caldiproteolyticus* SF03	Seletar, Singapore	Sewage sludge samples	Sewage sludge	Nutrient broth agar, tryptic soy agar, and plate count agar	A	35–65°C	60°C	6.0–9.0	6.5	-	-	AY327448
Clerck et al. [92]	*A. contaminans* LMG21881	France	French production plant	Gelatin samples	Nutrient agar supplemented with 1–2% gelatin	F. an	50–60°C	50°C	4.0–10.0	7.0	-	44.40%	NR_029006.1
Dulger et al. [96]	*A. kestanbolensis* K4	Çanakkale, Turkey	Hot spring	Water	Nutrient broth for enrichment	F. an	40–70°C	50–55°C	6.0–10.5	7.5–8.5	-	50%	AY248711
Yumoto et al. [119]	*A. voinovskiensis* TH3	Kamchatka, Russia	Hot spring	Water	TH agar medium, containing 5 g peptone, 3 g yeast extract, 5 g NaCl, 15 g agar, 3·5 mg EDTA, 3 mg ZnSO4.7H2O, 10 mg FeSO4.7H2O, 2 mg MnSO4.nH2O, 1 mg CuSO4.5H2O, 2 mg Co(NO3)2.6H2O and 1 mg H3BO3.	F. a	30–64°C	54°C	7.0–8.0	ND	-	43.90%	AB110008

Kevbrin et al. [102]	*A. kamchatkensis* JW/VK-KG4T	Kamchatka, Russia	Hot spring of the Valley of Geysers	Water sediment	Anaerobic mineral bicarbonate buffered medium	F. a	38–67°C	57–62°C	5.7–9.9	6.8–8.5	-	42.30%	AF510985
Poli et al. [10]	*A. amylolyticus* MR3C	Rittmann Mount, Antarctica	Geothermal soil	Geothermal soil	Enrichment media (A) containing Oxoid yeast extract (varying from 0.3% to 0.6%) and NaCl (0.3–0.6%) at pH 5.6 and pH 4.0	F. an	45–65°C	61°C	5.0–6.5	5.6	-	43.50%	AJ618979
Derekova et al. [95]	*A. rupienses* DSM 17127T	Rupi Basin, Bulgaria	Hot spring	Water and soil	PY medium (pH 7.0) containing peptone, 2 g l^{-1} and yeast extract, 1 g l^{-1}	A	35–67°C	55°C	5.5–8.5	6.0–6.5	-	41.70%	AJ879076
Atanassova et al. [84]	*A. bogrovensis* BT-13	Sofia, Bulgaria	Hot spring	Mud	PY medium (pH 7.0) containing (g 1^{-1}): peptone, 2 and yeast extract, 1<br>Plated on PY agar (pH 7.0)	F. an	40–69°C	65°C	6.5–10.0	8.0	-	44.10%	AM409184.1
Gul-Guven et al. [99]	*A. kamchatkensis* subsp. *asaccharedens* KG8	Batman, Turkey	Hot spring	Mud	Medium (C) nutrient broth	A	35–65°C	55°C	5.5–9.5	7.5	-	-	AM999779
Kacagan et al. [101]	*A. pushchinoensis* A8	Turkey	Hot spring	Mud and water	Nutrient agar	F. an	50–60°C	55°C	6.5–11.0	6.5	-	-	AY248715
Poli et al. [60]	*A. thermarum* AF/04	Padova, Italy	Hot spring	Thermal mud	Enrichment media (A) containing (% values are in w/v): peptone, 0.8%; yeast extract, 0.4%; and NaCl 0.2%	A	55–67°C	65°C	6.0–7.5	7.2	-	53.50%	AM402982
Namsaraev et al. [110]	*A. mongoliensis* T4	Arkhagai Aimag, Central Mongolia	Hot spring	Water sediment	Aerobic medium containing (g l^{-1}): KH_2PO_4, 0.5; NH_4Cl, 0.5; KCl, 0.5; NaCl, 0.5; Na_2SO_4, 0.5; $MgSO_4 \cdot 7H_2O$, 0.2; yeast extract, 1; peptone, 1; and glucose, 5; in addition	F. an	35–70°C	60°C	5.5–10.5	8.0	-	44%	EF654664

					to trace element solution, 1 ml								
Cihan et al. [90]	*A. salavatliensis* A343	Aydin, Turkey	Well pipeline sediment	Sediment	*Geobacillus thermoglucosidasius* medium (MI) containing 1% soluble starch (pH 7.0)	F. an	37–69°C	60°C	5.5–9.5	8.0–9.0	-	45.10%	EU326496
Gao et al. [98]	*Anoxybacillus* sp. PGDY12	Yunnan, China	Hot spring	Water sediment	LB medium supplemented with 0.05% toluene	A	65°C	55°C	5.0–10.0	7.0	-	-	FJ527829
Zhang et al. [120]	*A. eryuanensis* E-112	Yunnan, China	Hot spring	Water	LB medium	F. a	30–70°C	55°C	7.0–11.0	8.0	-	42.60%	GQ153549
Chai et al. [86]	*Anoxybacillus* sp. SK3-4	Sungai Klah, Malaysia	Hot spring	Water	Modified *Thermus* medium	A/An	30–65°C	55°C	6.0–10.0	7.0	-	42%	GQ184213
Zhang et al. [120]	*A. tengchongensis* T-11	Yunnan, China	Hot spring	Water	LB medium	F. a	30–75°C	50°C	7.0–11.0	8.5	-	41.10%	FJ438370
Coorevits et al. [95]	*A. caldiproteolyticus* R-35652	Singapore	Water reclamation plant	Sewage sludge	TSA	A	37–70°C	60°C	5.0–9.0	6.5–7.0	-	40.20%	NR_116989
Lee et al. [107]	*A. kamchatkensis* G10	Indonesia	Hot spring	-	-	F. an	-	-	-	-	2,962,568 bp	41.35%	ALJT01000000
Inan et al. [102]	*A. kaynarcensis* D1021	Izmir, Turkey	Hot spring	Water and mud	Nutrient broth for enrichment	A	35–70°C	60°C	6.0–10.0	7.0	-	42.90%	DSM 21706
Coorevits et al. [93]	*A. tepidamans* R-35643	Austria	Factory	Sugar beet extraction juice	TSA	F. an	40–65°C	55°C	6.0–9.0	5.0	-	43.20%	AY563003
Deep et al. [94]	*A. suryakundensis* JS1	Jharkhand, India	Hot spring	Sediment	TSA	F. an	40–60°C	55°C	5.5–11.5	7.5	-	42.10%	KC958552

Zhang et al. [120]	*A. vitaminiphilus* 3nP4	Sichuan, China	Hot spring	Water	Modified Brock medium, consisting of tryptone (3.0 g l^{-1}), yeast extract (3.0 g l^{-1}), and basic mineral medium	A	38–66°C	57–60°C	6.0–9.3	7.0–7.5	-	39.20%	FJ474084
Cihan et al. [91]	*A. calidus* C161ab	Denizli, Aegean Region, Turkey	Thermal power plant	Soil	*Geobacillus thermoglucosidasius* medium (MI) containing 1% soluble starch (pH 7.0)	F. an	35–70°C	55°C	6.5–9.0	8.0–8.5	-	37.80%	FJ430012
Matpan-Bekler and Guven [106]	*Anoxybacillus* sp. KP1	Ağri, Turkey	Hot spring	Mud and water	Nutrient broth medium	A	40–65°C	60°C	6.5–9.5	8.0	-	-	KC525949
Chen et al. [87]	*A. contaminans* HA	China	BioDeNOx treatment system	-	Modified denitrification medium (MDM)	A	30–60°C	55°C	-	-	-	-	KF973318
Patel [112]	*Anoxybacillus* sp. BC01	Australia	Great Artesian Basin (GAB)	Microbial mat colonization	Medium D	F. an	-	50°C	-	7.5	2.8 Mb	41.70%	JRLC01000000
Xia et al. [117]	*Anoxybacillus* sp. WJ-4	Longhu Pao	Daqing Oil Field	Brine and oil	Minimal medium supplemented with crude oil	F. a	45–80°C	68–72°C	-	6.0–8.0	-	44.30%	KR153283
Filippidou et al. [97]	*A. geothermalis* GSsed3	North Germany	Soil in geothermal factory	Soil	D2216 modified marine broth (This medium was modified by using 5 g tryptone instead of peptone, omitting the addition of potassium bromide, and adjusting the pH to 5.2 with HCl)	F. an	40–65°C	60°C	5.0–9.5	7.0–8.0	7,2 Mb	46.80%	JYCG00000000
Mittal et al. [108]	*A. mongoliensis* MB4	Surguja, Chhattisgarh, India	Hot spring	Water	Nutrient agar medium	-	-	-	-	-	30,188.3 bp	58.3%	MRZM01000000
Acer et al. [82]	*Anoxybacillus* sp. AH1	Ağri, Turkey	Hot spring	Water	Nutrient broth	A	40–70°C	55–60°C	6.0–11.0	9.0–10.0	-	-	KU997674

Chan et al. [77]	*Anoxybacillus* sp. DT3-1	Dusun Tua, Malaysia	Hot spring	Water	Modified *Thermus* medium	A/An	30–65°C	55°C	6.0–10.0	7.0	-	41.80%	GU129931
Al-Kahem Al-Balawi et al. [83]	*Anoxybacillus* sp. UARK-01	Arkansas, USA	Soil beneath grass	Soil	Media containing mineral salts and 0.5% (w/v) switchgrass as the sole carbon source	A	50–60°C	55°C	7.0–9.0	8.0	3,669,492 bp	42.60%	PRJNA379989
Yadav et al. [118]	*A. kamchatkensis* NASTPD13	Myagdi, Nepal	Hot spring	Water	Minimal salt medium (MSM)	F. an	37–75°C	-	5.0–11.0	-	-	-	KY373247
Khan et al. [103]	*A. sediminis* YIM 73012	Tibet, China	Qucai geothermal field	Sediment in geothermal site	R2A agar medium	A	37–65°C	45–50°C	6.0–8.5	7.0–7.5	-	43.60%	MG661737
Matpan-Bekler et al. [107]	*Anoxybacillus sp.* FMB1	Yozgat, Turkey	Hot spring	Water	Modified liquid *Thermus* medium	A	25–40°C	50°C	4.0–11.0	7.0	-	-	KP992869
Reis et al. [113]	*A. sp.* PC2	Caatinga, Brazil	Caatinga	Soil	Enrichment culture medium	F. an	50–60°C	50–60°C	5.0–10.0	7.0	-	-	MN937679
Cheng et al. [89]	*A. caldiproteolyticus* 1A02591	East of the Pacific Ocean	Deep sea hydrotherm al vent	Sediment	2216E medium (0.5% Bacto peptone, 0.1% yeast extract, and artificial seawater; pH 7.5)	A	45–65°C	55°C	-	-	-	-	JAEILW0000000 00

*** A = Aerobe; An = Anaerobe; F. a = Facultative aerobe; F. an = Facultative anaerobe**

Table S3. Metagenomic data on *Anoxybacillus*, retrieved from public databases

Reference	Species	Location	Source	Substrate
Khanna et al. [104]	*Anoxybacillus* sp.	-	Rat	Fecal samples
Najar et al. [109]	*A. gonensis* and *A. caldiproteolyticus*	Borong and Polok hot springs of South Sikkim, India	Hot springs	Water and soil
Ottesen et al. [111]	*Anoxybacillus* sp.	USA	Ice cream scoops	Ice cream

Sahm et al. [114]	*Anoxybacillus* sp.	Furnas Valley, Sao Miguel, Azores, Portugal	Hot spring	Sediment, biofilm, and water
Salam and Obayori [115]	*Anoxybacillus* sp.	Ilorin, Kwara State, Nigeria	Composite animal charcoal-polluted soil	Soil
Wu et al. [116]	*Anoxybacillus* sp.	Yangtze River, China	River and fish	Sediment, fish intestinal content, and fish mucosa
Zhao et al. [122]	*A. flavithermus*	Xiamen, China	Hot spring	Sediment

Table S4. Regions of phages in the Antarctic *Anoxybacillus flavithermus* strains isolated in the present study, as predicted using PHASTER

Genomes	Region	Region Length	Completeness	Score	Total Proteins	Region Position	Most common phage	G + C content
**LAT_11**	1 - NODE_1_length_418483_cov_331.812911	24.4 Kb	Incomplete	40	17	11894–36301	PHAGE_Bacill_phiNIT1_NC_021856(2)	39.69%
	2 - NODE_2_length_445795_cov_336.731076	24.4 Kb	Incomplete	40	17	11894–36301	PHAGE_Staphy_SPbeta_like_NC_029119(2)	39.69%
**LAT_26**	1 - 04-edena/D136_FSFP192285480-1a_HWNCGDSXX_L2__30	5.3 Kb	Incomplete	20	9	3–5358	PHAGE_Strept_Dp_1_NC_015274(2)	42.38%
	2 - NODE_18_length_49609_cov_303.747719	33.6 Kb	Intact	140	50	15746–49378	PHAGE_Thermu_OH2_NC_021784(26)	41.44%

	3 - NODE_1_length_650285_cov_263.707080	24.3 Kb	Incomplete	40	17	614060–638453	PHAGE_Bacill_phiNIT1_NC_021856(2)	39.68%
	4 - NODE_4_length_130475_cov_266.489065	43.7 Kb	Intact	130	55	350–44128	PHAGE_Thermu_OH2_NC_021784(12)	41.32%
	5 - NODE_1_length_649997_cov_270.183562	24.3 Kb	Incomplete	40	17	613772–638165	PHAGE_Bacill_phiNIT1_NC_021856(2)	39.68%
**LAT_27**	1 - NODE_1_length_456493_cov_294.959721	24.4 Kb	Incomplete	40	17	11898–36305	PHAGE_Staphy_SPbeta_like_NC_029119(2)	39.69%
**LAT_31**	1 - NODE_1_length_457267_cov_52.1925_component_0	24.3 Kb	Incomplete	40	17	420909–445302	PHAGE_Staphy_SPbeta_like_NC_029119(2)	39.68%
	2 - NODE_10_length_95172_cov_50.4712_component_0	4.8 Kb	Incomplete	20	9	2–4805	PHAGE_Bacill_BCD7_NC_019515(2)	42.61%
**LAT_33**	1 - NODE_1_length_457267_cov_52.1925_component_0	5.1 Kb	Incomplete	20	9	89034–94170	PHAGE_Bacill_BCD7_NC_019515(2)	42.52%
	2 - 04-edena/D144_FSFP192285488-1a_HWNCGDSXX_L3__12,size=120848,cov=496.29	24.4 Kb	Incomplete	40	17	12519–36926	PHAGE_Staphy_SPbeta_like_NC_029119(2)	39.69%
**LAT_35**	1 - 04-edena/D146_FSFP192285490-1a_HWNCGDSXX_L3__1,size=120698,cov=597.479	24.4 Kb	Incomplete	40	17	12369–36776	PHAGE_Bacill_phiNIT1_NC_021856(2)	39.69%
	2 - 04-edena/D146_FSFP192285490-1a_HWNCGDSXX_L3__37,size=18327,cov=588.787	5.1 Kb	Incomplete	20	9	3–5139	PHAGE_Bacill_BCD7_NC_019515(2)	42.52%

**LAT_38**	1 - 04-edena/D149_FDMS192285493-1a_HWNCGDSXX_L3__1	5.6 Kb	Incomplete	20	9	1–5698	PHAGE_Strept_Dp_1_NC_015274(2)	42.01%
	2 - NODE_18_length_49621_cov_333.261575	33.6 Kb	Intact	140	50	15752–49384	PHAGE_Thermu_OH2_NC_021784(26)	41.44%
	3 - NODE_1_length_501254_cov_426.879991	24.4 Kb	Incomplete	40	17	11825–36232	PHAGE_Staphy_SPbeta_like_NC_029119(2)	39.69%
	4 - NODE_2_length_272886_cov_474.757143	43.7 Kb	Intact	150	59	356–44134	PHAGE_Geobac_E2_NC_009552(13)	41.33%
	5 - NODE_17_length_49621_cov_333.261575	33.6 Kb	Intact	140	50	15752–49384	PHAGE_Thermu_OH2_NC_021784(26)	41.44%

Table S5. Genes related to the lifestyle of *Anoxybacillus flavithermus* strains and their corresponding locus tag, annotated with GO FEAT

	**Genes/Locus tag (when present)**																						
**Genomes**	**DnaK**	**DnaJ**	**GrpE**	**Hsp20**	***hcr*A**	**ClpC**	**ClpP**	***yfl*T**	***htp*X**	**GroEL**	**GroES**	**CspB**	**CspD**	**Na+/H+ antiporter subunits A−G**	***nha*C**	**UvrA**	**UvrB**	**UVrC**	***Mut*S**	***Mut*L**	***Lig*A**	***Rec*R**	**RuV**
**LAT_11**	N_01457	NFPJCLFB_01458	NFPJCLFB_01456	NFPJCLFB_02018	-	NFPJCLFB_00547	NFPJCLFB_00387<br>NFPJCLFB_02866	-	NFPJCLFB_00200	-	-	NFPJCLFB_00102	NFPJCLFB_00325<br>NFPJCLFB_01003	NFPJCLFB_01143<br>NFPJCLFB_01144	-	NFPJCLFB_02465<br>NFPJCLFB_02841<br>NFPJCLFB_02842<br>NFPJCLFB_03122	NFPJCLFB_02841	NFPJCLFB_02465<br>NFPJCLFB_03122	NFPJCLFB_00065<br>NFPJCLFB_02481<br>NFPJCLFB_02483<br>NFPJCLFB_03138<br>NFPJCLFB_03140	NFPJCLFB_00066	NFPJCLFB_00002<br>NFPJCLFB_00070<br>NFPJCLFB_00073<br>NFPJCLFB_00080<br>NFPJCLFB_00183<br>NFPJCLFB_00427<br>NFPJCLFB_00428<br>NFPJCLFB_00478<br>NFPJCLFB_00540<br>NFPJCLFB_00542*	NFPJCLFB_00998	NFPJCLFB_00017<br>NFPJCLFB_00018<br>NFPJCLFB_00113<br>NFPJCLFB_00758<br>NFPJCLFB_00783<br>NFPJCLFB_00996<br>NFPJCLFB_01048<br>NFPJCLFB_01121<br>NFPJCLFB_01150<br>NFPJCLFB_01151*

**LAT_26**	MKDEMENN_02047<br>MKDEMENN_03406	MKDEMENN_02046<br>MKDEMENN_03405	MKDEMENN_02048<br>MKDEMENN_03407	MKDEMENN_00384<br>MKDEMENN_01353	-	ILCLCDJK_00598	MKDEMENN_02382	-	MKDEMENN_01271	-	-	MKDEMENN_00017	MKDEMENN_00238<br>MKDEMENN_01105	MKDEMENN_00867<br>MKDEMENN_00868<br>MKDEMENN_03677<br>MKDEMENN_03678	-	MKDEMENN_00360<br>MKDEMENN_02101<br>MKDEMENN_02406<br>MKDEMENN_02407<br>MKDEMENN_03460	MKDEMENN_02407	MKDEMENN_02101<br>MKDEMENN_03460	MKDEMENN_00054<br>MKDEMENN_02083<br>MKDEMENN_02085<br>MKDEMENN_03442<br>MKDEMENN_03444	MKDEMENN_00053	MKDEMENN_00039<br>MKDEMENN_00046<br>MKDEMENN_00049<br>MKDEMENN_00116<br>MKDEMENN_00408<br>MKDEMENN_00409<br>MKDEMENN_00459<br>MKDEMENN_00521<br>MKDEMENN_00523<br>MKDEMENN_00576*	MKDEFPJCLFBMENN_01059	MKDEMENN_01059
**LAT_27**	AOKPIELD_02683	AOKPIELD_02682	AOKPIELD_02684	AOKPIELD_00070<br>AOKPIELD_00493<br>AOKPIELD_02246	-	AOKPIELD_02402	AOKPIELD_01161<br>AOKPIELD_02018	-	AOKPIELD_00838	-	-	AOKPIELD_00017<br>AOKPIELD_00630	AOKPIELD_00321<br>AOKPIELD_01299	AOKPIELD_00805<br>AOKPIELD_00806	-	AOKPIELD_02042<br>AOKPIELD_02043<br>AOKPIELD_02926	AOKPIELD_02042<br>AOKPIELD_02043<br>AOKPIELD_02926	AOKPIELD_02926	AOKPIELD_00667<br>AOKPIELD_02942<br>AOKPIELD_02944	AOKPIELD_00666	AOKPIELD_00039<br>AOKPIELD_00046<br>AOKPIELD_00049<br>AOKPIELD_00079<br>AOKPIELD_00082<br>AOKPIELD_0010	AOKPIELD_00183	AOKPIELD_00006<br>AOKPIELD_00181<br>AOKPIELD_00270<br>AOKPIELD_00619<br>AOKPIELD_00714<br>AOKPIELD_

																					9<br>AOKPIELD_00116<br>AOKPIELD_00117<br>AOKPIELD_00176<br>AOKPIELD_00223*		00715<br>AOKPIELD_00730<br>AOKPIELD_00752<br>AOKPIELD_00753<br>AOKPIELD_00754*
**LAT_31**	INBDBCKJ_00691	INBDBCKJ_00690	INBDBCKJ_00692	INBDBCKJ_00748<br>INBDBCKJ_01997	-	INBDBCKJ_02122	INBDBCKJ_01132<br>INBDBCKJ_01424	-	INBDBCKJ_01772	-	INBDBCKJ_02716	INBDBCKJ_01253	INBDBCKJ_01074<br>INBDBCKJ_02792	INBDBCKJ_01614<br>INBDBCKJ_01615	-	INBDBCKJ_00291<br>INBDBCKJ_01399<br>INBDBCKJ_01400	INBDBCKJ_01399	INBDBCKJ_00291	INBDBCKJ_00273<br>INBDBCKJ_00275<br>INBDBCKJ_01216	INBDBCKJ_01217	INBDBCKJ_00038<br>INBDBCKJ_00085<br>INBDBCKJ_00094<br>INBDBCKJ_00109<br>INBDBCKJ_00112<br>INBDBCKJ_00125<br>INBDBCKJ_00137<br>INBDBCKJ_00142<br>INBDBCKJ_00189<br>INBDBCKJ_00242 *	INBDBCKJ_02724	INBDBCKJ_00035<br>INBDBCKJ_00074<br>INBDBCKJ_00168<br>INBDBCKJ_00374<br>INBDBCKJ_00375<br>INBDBCKJ_00559<br>INBDBCKJ_00672<br>INBDBCKJ_00988<br>INBDBCKJ_01169<br>INBDBCKJ_01264*

**LAT_33**	ANMPAFOB_02765	ANMPAFOB_02766	ANMPAFOB_02764	ANMPAFOB_00546<br>ANMPAFOB_02225	-	ANMPAFOB_01441	ANMPAFOB_00745<br>ANMPAFOB_01713	-	ANMPAFOB_00083	-	ANMPAFOB_02633	ANMPAFOB_00209	ANMPAFOB_01771<br>ANMPAFOB_02683	ANMPAFOB_00165<br>ANMPAFOB_00166	-	ANMPAFOB_00769<br>ANMPAFOB_00770<br>ANMPAFOB_00875	ANMPAFOB_00770	ANMPAFOB_00875	ANMPAFOB_00875	ANMPAFOB_00245	ANMPAFOB_00066<br>ANMPAFOB_00189<br>ANMPAFOB_00238<br>ANMPAFOB_00241<br>ANMPAFOB_00301<br>ANMPAFOB_00526<br>ANMPAFOB_00528<br>ANMPAFOB_00535<br>ANMPAFOB_00826<br>ANMPAFOB_00827*	ANMPAFOB_02641	ANMPAFOB_00112<br>ANMPAFOB_00113<br>ANMPAFOB_00114<br>ANMPAFOB_00157<br>ANMPAFOB_00158<br>ANMPAFOB_00159<br>ANMPAFOB_00198<br>ANMPAFOB_00348<br>ANMPAFOB_00687<br>ANMPAFOB_00697<br>*
**LAT_35**	LBOCMBFH_01052	LBOCMBFH_01051	LBOCMBFH_01053	LBOCMBFH_02160	-	LBOCMBFH_02066	LBOCMBFH_00213<br>LBOCMBFH_01797	-	LBOCMBFH_00778	-	LBOCMBFH_02607	LBOCMBFH_01380	LBOCMBFH_01281<br>LBOCMBFH_02513	LBOCMBFH_02326<br>LBOCMBFH_02327	-	LBOCMBFH_00237<br>LBOCMBFH_00238<br>LBOCMBFH_01637	LBOCMBFH_00238	LBOCMBFH_01637	LBOCMBFH_00297<br>LBOCMBFH_00299<br>LBOCMBFH_02092	LBOCMBFH_02091	LBOCMBFH_00076<br>LBOCMBFH_00089<br>LBOCMBFH_00090<br>LBOCMBFH_00295<br>LBOCMBFH_00311<br>LBOCM	LBOCMBFH_02494	LBOCMBFH_00107<br>LBOCMBFH_00108<br>LBOCMBFH_00155<br>LBOCMBFH_00165<br>LBOCMBFH_00394<br>LBOC

																					BFH_00319 LBOCMBFH_00320 LBOCMBFH_00373 LBOCMBFH_00420 LBOCMBFH_00425*		MBFH_00908 LBOCMBFH_01033 LBOCMBFH_01364 LBOCMBFH_01369 LBOCMBFH_01536*
**LAT_38**	ILCLCDJK_01998	ILCLCDJK_01999	ILCLCDJK_01997	ILCLCDJK_00501 ILCLCDJK_01512	-	ILCLCDJK_00598	ILCLCDJK_02415	-	ILCLCDJK_00589	-	-	ILCLCDJK_00219	ILCLCDJK_00355 ILCLCDJK_01283	ILCLCDJK_00027 ILCLCDJK_00028	-	ILCLCDJK_00477 ILCLCDJK_01944 ILCLCDJK_02439 ILCLCDJK_02440	ILCLCDJK_02440	ILCLCDJK_01944	ILCLCDJK_00256 ILCLCDJK_01960 ILCLCDJK_01962	ILCLCDJK_00255	ILCLCDJK_00004 ILCLCDJK_00129 ILCLCDJK_00135 ILCLCDJK_00140 ILCLCDJK_00142 ILCLCDJK_00163 ILCLCDJK_00183 ILCLCDJK_00241 ILCLCDJK_00248 ILCLCDJK_00251*	ILCLCDJK_01253	ILCLCDJK_00005 ILCLCDJK_00034 ILCLCDJK_00035 ILCLCDJK_00036 ILCLCDJK_00079 ILCLCDJK_00080 ILCLCDJK_00081 ILCLCDJK_00103 ILCLCDJK_00145 ILCLCDJK_00208*

*: More than 10 locus tags in the corresponding gene.

Table S6. Methodologies applied in previous related phylogenetic studies

Phylogeny/ Phylogenomics	Methodology	Gene	Reference
Phylogenetic analysis of 16S rRNA	Neighbor-joining method	16S rRNA	Acer Ö, Bekler FM, Pirinççioğlu H, Güven RG, Güven K. Purification and characterization of thermostable and detergent-stable α-amylase from *Anoxybacillus* sp. AH1. Food Technol Biotechnol. **2016**, *54*, 70-77. doi: 10.17133/ftb.54.01.16.4122. PMID: 27904395. PMCID: PMC5105632.
Phylogenetic analysis of 16S rRNA	Neighbor-joining and maximum likelihood methods	16S rRNA	Al-Kahem Al-Balawi TH, Wood AL, Solis A, Cooper T, Barabote RD. *Anoxybacillus* sp. strain UARK-01, a new thermophilic soil bacterium with hyperthermostable alkaline laccase activity. Curr Microbiol. **2017**, *74*, 762-771. doi: 10.1007/s00284-017-1239-5. Epub 2017 Apr 8. PMID: 28389772.
Phylogenetic analysis of 16S rRNA	Maximum likelihood method	16S rRNA	Atanassova M, Derekova A, Mandeva R, Sjøholm C, Kambourova M. *Anoxybacillus bogrovensis* sp. nov., a novel thermophilic bacterium isolated from a hot spring in Dolni Bogrov, Bulgaria. Int J Syst Evol Microbiol. **2008** *58*, 2359-2362. doi: 10.1099/ijs.0.65745-0. PMID: 18842856.
Phylogenetic analysis of 16S rRNA	Neighbor-joining method	16S rRNA	Matpan-Bekler F, Guven K. Isolation and production of thermostable α-amylase from thermophilic *Anoxybacillus* sp. KP1 from Diyadin hot spring in Ağri, Turkey. Biologia. **2014**, *69*, 419-427. doi: 10.2478/s11756-014-0343-2.
Phylogenetic analysis of 16S rRNA	Neighbor-joining method	16S rRNA	Matpan-Bekler F, Yalaz S, Guven K. Molecular characterisation and numerical analysis of novel moderately thermophile *Anoxybacillus* sp. FMB1. Rom Biotechnol Lett. **2018**, *23*, 13964-13975. doi: 10.26327/RBL2018.150.
Phylogenetic analysis of 16S rRNA	Neighbor-joining method	16S rRNA	Chai YY, Kahar UM, Md Salleh M, Md Illias R, Goh KM. Isolation and characterization of pullulan-degrading *Anoxybacillus* species isolated from Malaysian hot springs. Environ Technol. **2012**, *33*, 1231-1238. doi: 10.1080/09593330.2011.618935. PMID: 22856294.
Phylogenetic analysis of DT-Bgl and other β-glucosidases	Neighbor-joining method	DT-Bgl and other β-glucosidases	Chan CS, Sin LL, Chan KG, Shamsir MS, Manan FA, Sani RK, Goh KM. Characterization of a glucose-tolerant β-glucosidase from *Anoxybacillus* sp. DT3-1. Biotechnol Biofuels. **2016**, *9*, 174. doi: 10.1186/s13068-016-0587-x. PMID: 27555880. PMCID: PMC4994278.
Phylogenetic analysis of 16S rRNA	Maximum likelihood method	16S rRNA	Chen XG, Stabnikova O, Tay JH, Wang JY, Tay ST. Thermoactive extracellular proteases of *Geobacillus caldoproteolyticus* sp. nov., from sewage sludge. Extremophiles. **2004,** *8*, 489-498. doi: 10.1007/s00792-004-0412-5. Epub 2004 Aug 21. PMID: 15322950.

Phylogenetic analysis of 16S rRNA	Neighbor-joining method	16S rRNA	Chen J, Zheng J, Li Y, Hao HH, Chen JM. Characteristics of a novel thermophilic heterotrophic bacterium, *Anoxybacillus contaminans* HA, for nitrification-aerobic denitrification. Appl Microbiol Biotechnol. **2015**, *99*, 10695-10702. doi: 10.1007/s00253-015-6870-0. Epub 2015 Aug 21. PMID: 26293335.
Phylogenetic analysis of 16S rRNA	Maximum likelihood method	16S rRNA	Cihan AC, Ozcan B, Cokmus C. *Anoxybacillus salavatliensis* sp. nov., an α-glucosidase producing, thermophilic bacterium isolated from Salavatli, Turkey. J Basic Microbiol. **2011**, *51*, 136-146. doi: 10.1002/jobm.201000115. Epub 2010 Nov 12. PMID: 21077116.
Phylogenetic analysis of 16S rRNA	Neighbor-joining method	16S rRNA	Cihan AC, Cokmus C, Koc M, Ozcan B. *Anoxybacillus calidus* sp. nov., a thermophilic bacterium isolated from soil near a thermal power plant. Int J Syst Evol Microbiol. **2014,** *64*, 211-219. doi: 10.1099/ijs.0.056549-0. Epub 2013 Sep 19. Erratum in: Int J Syst Evol Microbiol. 2014 Aug;64(Pt 8):2926. Erratum in: Int J Syst Evol Microbiol. 2014 Jul;64(Pt 7):2508. PMID: 24052627.
Phylogenetic analysis of 16S rRNA	Maximum likelihood method	16S rRNA	Coorevits A, Dinsdale AE, Halket G, Lebbe L, De Vos P, Van Landschoot A, Logan NA. Taxonomic revision of the genus Geobacillus: emendation of *Geobacillus*, *G. stearothermophilus*, *G. jurassicus*, *G. toebii*, *G. thermodenitrificans* and *G. thermoglucosidans* (nom. corrig., formerly '*thermoglucosidasius*'); transfer of *Bacillus thermantarcticus* to the genus as *G. thermantarcticus* comb. nov.; proposal of *Caldibacillus debilis* gen. nov., comb. nov.; transfer of *G. tepidamans* to *Anoxybacillus* as *A. tepidamans* comb. nov.; and proposal of *Anoxybacillus caldiproteolyticus* sp. nov. Int J Syst Evol Microbiol. **2012,** *62*, 1470-1485. doi: 10.1099/ijs.0.030346-0. Epub 2011 Aug 19. PMID: 21856988.
Phylogenetic analysis of 16S rRNA	Neighbor-joining method	16S rRNA	Deep K, Poddar A, Das SK. *Anoxybacillus suryakundensis* sp. nov, a moderately thermophilic, alkalitolerant bacterium isolated from hot spring at Jharkhand, India. PLoS One. **2013** *20*;8(12):e85493. doi: 10.1371/journal.pone.0085493. PMID: 24376881. PMCID: PMC3869905.
Phylogenetic analysis of 16S rRNA	Neighbor-joining and maximum likelihood methods	16S rRNA	Derekova A, Sjøholm C, Mandeva R, Kambourova M. *Anoxybacillus rupiensis* sp. Nov., a novel thermophilic bacterium isolated from Rupi basin (Bulgaria). Extremophiles. **2007**, *11*, 577-583. doi: 10.1007/s00792-007-0071-4. Epub 2007 May 16. PMID: 17505776.
Phylogenetic analysis of 16S rRNA	Neighbor-joining method	16S rRNA	Dulger S, Demirbag Z, Belduz AO. *Anoxybacillus ayderensis* sp. nov. and *Anoxybacillus kestanbolensis* sp. nov. Int J Syst Evol Microbiol. **2004**, *54*, 1499-1503. doi: 10.1099/ijs.0.02863-0. PMID: 15388701.

Phylogenetic analysis of 16S rRNA	Neighbor-joining method	16S rRNA	Filippidou S, Jaussi M, Junier T, Wunderlin T, Jeanneret N, Palmieri F, Palmieri I, Roussel-Delif L, Vieth-Hillebrand A, Vetter A, Chain PS, Regenspurg S, Junier P. *Anoxybacillusgeothermalis* sp. nov., a facultatively anaerobic, endospore-forming bacterium isolated from mineral deposits in a geothermal station. Int J Syst Evol Microbiol. **2016,** *66*, 2944-2951. doi: 10.1099/ijsem.0.001125. Epub 2016 Apr 28. PMID: 27126386.
Phylogenetic analysis of 16S rRNA	Neighbor-joining method	16S rRNA	Gao Y, Dai J, Peng H, Liu Y, Xu T. Isolation and characterization of a novel organic solvent-tolerant *Anoxybacillus* sp. PGDY12, a thermophilic gram-positive bacterium. J Appl Microbiol. **2011**, *110*, 472-478. doi: 10.1111/j.1365-2672.2010.04903.x. Epub 2010 Dec 13. PMID: 21155953.
Phylogenetic analysis of 16S rRNA	Neighbor-joining method	16S rRNA	Gul-Guven R, Guven K, Poli A, Nicolaus B. *Anoxybacillus kamchatkensis* subsp. *asaccharedens* subsp. nov., a thermophilic bacterium isolated from a hot spring in Batman. J Gen Appl Microbiol. **2008**, *54*, 327-334. doi: 10.2323/jgam.54.327. PMID: 19164875.
Phylogenetic analysis of 16S rRNA	Neighbor-joining method	16S rRNA	Inan K, Belduz AO, Canakci S. *Anoxybacillus kaynarcensis* sp. nov., a moderately thermophilic, xylanase producing bacterium. J Basic Microbiol. **2013**, *53*, 410-419. doi: 10.1002/jobm.201100638. Epub 2012 Jun 26. PMID: 22736500.
Phylogenetic analysis of 16S rRNA	Neighbor-joining method	16S rRNA	Kevbrin VV, Zengler K, Lysenko AM, Wiegel J. *Anoxybacillus kamchatkensis* sp. nov., a novel thermophilic facultative aerobic bacterium with a broad pH optimum from the Geyser valley, Kamchatka. Extremophiles. **2005** *9*, 391-398. doi: 10.1007/s00792-005-0479-7. Epub 2005 Sep 3. PMID: 16142505.
Phylogenetic analysis of 16S rRNA	Neighbor-joining method	16S rRNA	Khan IU, Habib N, Xiao M, Devi AM, Habib M, Hejazi MS, Salam N, Zhi XY, Li WJ. *Anoxybacillus sediminis* sp. nov., a novel moderately thermophilic bacterium isolated from a hot spring. Antonie Van Leeuwenhoek. **2018**, *111*, 2275-2282. doi: 10.1007/s10482-018-1118-5. Epub 2018 Jun 21. PMID: 29931494.
Phylogenetic analysis of 16S rRNA	Neighbor-joining method	16S rRNA	Namsaraev ZB, Babasanova OB, Dunaevsky YE, Akimov VN, Barkhutova DD, Gorlenko VM, Namsaraev BB. *Anoxybacillus mongoliensis* sp. nov., a novel thermophilic proteinase producing bacterium isolated from alkaline hot spring, Central Mongolia. Mikrobiologiia. **2010** *79*, 516-523. PMID: 21058505.
Phylogenetic analysis of 16S rRNA	Neighbor-joining method	16S rRNA	Poli A, Esposito E, Lama L, Orlando P, Nicolaus G, de Appolonia F, Gambacorta A, Nicolaus B. *Anoxybacillus amylolyticus* sp. nov., a thermophilic amylase producing bacterium isolated from Mount Rittmann (Antarctica). Syst Appl Microbiol. **2006,** *29*, 300-307. doi: 10.1016/j.syapm.2005.10.003. Epub 2005 Nov 3. PMID: 16682297.

Phylogenetic analysis of 16S rRNA	Neighbor-joining method	16S rRNA	Poli A, Romano I, Cordella P, Orlando P, Nicolaus B, Ceschi Berrini C. *Anoxybacillus thermarum* sp. nov., a novel thermophilic bacterium isolated from thermal mud in Euganean hot springs, Abano Terme, Italy. Extremophiles. **2009**, *13*, 867-874. doi: 10.1007/s00792-009-0274-y. Epub 2009 Aug 27. PMID: 19710998.
Phylogenetic analysis of 16S rRNA	Determined by the neighbor-joining algorithm and evaluated by maximum likelihood and maximum parsimony algorithms	16S rRNA	Xia W, Dong H, Zheng C, Cui Q, He P, Tang Y. Hydrocarbon degradation by a newly isolated thermophilic *Anoxybacillus* sp. with bioemulsifier production and new alkB genes. RSC Adv. **2015**, *5*, 102367-102377. doi: 10.1039/C5RA17137G.
Phylogenetic analysis of 16S rRNA	Maximum likelihood method	16S rRNA	Yadav P, Maharjan J, Korpole S, Prasad GS, Sahni G, Bhattarai T, Sreerama L. Production, purification, and characterization of thermostable alkaline xylanase from *Anoxybacillus kamchatkensis* NASTPD13. Front Bioeng Biotechnol. **2018**, *6*:65. doi: 10.3389/fbioe.2018.00065
Phylogenetic analysis of 16S rRNA	Neighbor-joining method	16S rRNA	Yumoto I, Hirota K, Kawahara T, Nodasaka Y, Okuyama H, Matsuyama H, Yokota Y, Nakajima K, Hoshino T. *Anoxybacillus voinovskiensis* sp. nov., a moderately thermophilic bacterium from a hot spring in Kamchatka. Int J Syst Evol Microbiol. **2004**, *54*, 1239-1242. doi: 10.1099/ijs.0.02889-0. PMID: 15280298.
Phylogenetic analysis of 16S rRNA	Neighbor-joining method	16S rRNA	Zhang CM, Huang XW, Pan WZ, Zhang J, Wei KB, Klenk HP, Tang SK, Li WJ, Zhang KQ. *Anoxybacillus tengchongensis* sp. nov. and *Anoxybacillus eryuanensis* sp. nov., facultatively anaerobic, alkalitolerant bacteria from hot springs. Int J Syst Evol Microbiol. **2011,** *61*, 118-122. doi: 10.1099/ijs.0.020834-0. Epub 2010 Feb 19. PMID: 20173008.
Phylogenetic analysis of 16S rRNA	Neighbor-joining method	16S rRNA	Zhang XQ, Zhang ZL, Wu N, Zhu XF, Wu M. *Anoxybacillus vitaminiphilus* sp. nov., a strictly aerobic and moderately thermophilic bacterium isolated from a hot spring. Int J Syst Evol Microbiol. **2013,** *63*, 4064-4071. doi: 10.1099/ijs.0.050096-0. Epub 2013 May 31. PMID: 23728374.

Table S7. Details of strains used in genomic analysis

**Name**	**NCBI Ref Seq Assembly Accession**	**Status**
Geobacillus kaustophilus strain HTA426	GCF_000009785.1	Complete genome
Geobacillus genomo sp. 3 JF8	GCF_000445995.2	Complete genome
Geobacillus sp. 12AMOR1	GCF_001028085.1	Complete genome
Brevibacillus brevis DZQ7	GCF_001039275.2	Complete genome
Brevibacillus brevis NBRC 100599	GCF_000010165.1	Complete genome
Anoxybacillus amylolyticus DSM 15939	GCF_001634285.1	Complete genome
Anoxybacillus sp. B7M1	GCF_001634305.1	Complete genome
Anoxybacillus flavithermus WK1	GCF_000019045.1	Complete genome
